# Osteosarcoma: A Comparison of Jaw versus Nonjaw Localizations and Review of the Literature

**DOI:** 10.1155/2013/316123

**Published:** 2013-07-15

**Authors:** H. van den Berg, W. H. Schreuder, J. de Lange

**Affiliations:** ^1^Department of Pediatric Oncology, Emma Children Hospital Academic Medical Centre, University of Amsterdam, Room G8-147, P.O. Box 22700, 1100 DD Amsterdam, The Netherlands; ^2^Medicines Evaluation Board, Utrecht, The Netherlands; ^3^Department of Oral and Maxillofacial Surgery, Academic Medical Centre, University of Amsterdam, The Netherlands

## Abstract

*Purpose*. It is assumed that osteosarcomas of the jaws mainly occur at older ages, whereas the most prominent sites, that is, the long bones, are more affected at ages <20. Jaw-localized tumors are less malignant and have lower metastatic spread rates. *Patients and Methods*. This study analyses the nationwide data of the Dutch Cancer Registry on osteosarcoma during the period from 1991 to 2010. Age-corrected incidence rates were calculated. *Results*. In 949, 38 patients had tumors in the maxilla and in 58 in the mandible. Median age for maxilla, mandible, and other localizations was 45.5, 49, and 23 years, respectively. Age-corrected incidence for osteosarcomas increased after a steep decline for the age cohorts from 20 to 60 years to nearly the same level as the younger patients. The incidence for maxillary lesions showed a steady increase from 0.46 to 1.60 per million over all age ranges; the highest incidence for mandibular lesions was found in the age cohort from 60 to 79 years. In respect to histology, no shifts for age were found, except for Paget's disease-related osteosarcoma. In older patients, chemotherapy was omitted more often. Overall survival was similar for all age groups, except for extragnatic tumor patients in the age range of 60–79 years. *Conclusions*. Osteosarcomas have comparable incidences below the age of 20 as compared with ages >60 years. Poorer outcome in older people is likely due to refraining from chemotherapy.

## 1. Introduction

Osteosarcoma is the most common malignancy of bone. Based on location of the tumor differences in occurrence, clinical behavior and outcome are assumed. This is especially the case for osteosarcoma of the maxilla and mandible. These locations are presumed to be rare. Percentages of 1 to 9% of the total number of osteosarcomas are mentioned [1–5]. It is further claimed that the mean age of presentation of craniofacial osteosarcomas is at least 10 to 15 years later than osteosarcomas in other parts of the body. However, reports are based on data sources from single institutions or are compiled from registries [4, 6–11]. It is further claimed that tumors in mandible and maxilla are less malignant as based on more often occurrence of low malignant histology and in particular on clinical outcome, that is, a better event, and overall prognosis, and lower incidence of metastatic spread as compared with osteosarcomas arising elsewhere in the body. Metastatic spread at initial presentation up to 16% is reported [3, 7, 11–16]. In this paper the nationwide data on osteosarcomas, as registered by the Netherlands, Cancer Registry of the Comprehensive Cancer Centers of the Netherlands are described.

## 2. Methods

The retrospective study was carried out on basis of the nationwide coverage of the Netherlands Cancer Registry over the period 1991–2010. Data that could be collected were year of diagnosis, age at diagnosis, localization, histological type of osteosarcoma, occurrence of death, duration of followup, and type of treatment (surgery, radiotherapy, and chemotherapy). Calculations on incidence were done on basis of population data, as obtained from the Statline database of Statistics Netherlands. Statistics Netherlands, a governmental institution, is responsible for collecting and processing population data in order to publish statistics to be used in practice, by policymakers and for scientific research (http://www.cbs.nl/). For statistical analysis, the SPSS version 19.0 was used. Approval of this retrospective study of blinded data was not needed by law. 

## 3. Results

**Figure 1:**
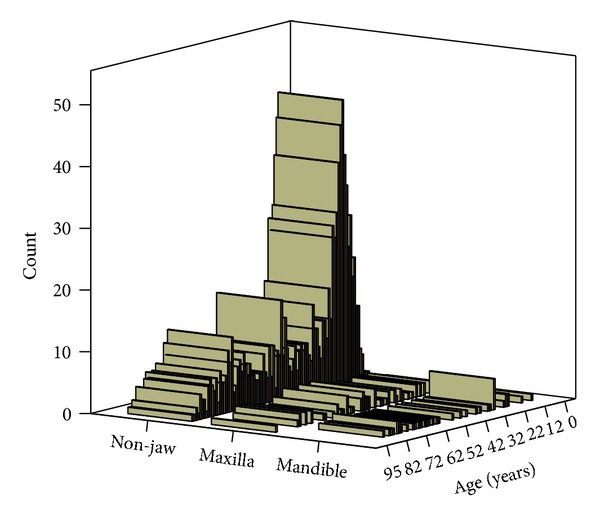
Number of patients per age.

In total, 949 patients were registered, that is, 499 males and 450 females. In 96 patients, the osteosarcoma was localized in the mandible (*n* = 38, 4%) or in the maxilla, inclusive adjacent bones (*n* = 58, 6%). Ages ranged from 0 to 95 years, median 25.00 years and mean 34.68 years. For nonfacial bones ages ranged from 0 to 92 years, median 23.00 and mean 33.52 years, for maxilla and adjacent bones ages ranged from 3 to 92 years, median 45.50 and mean 44.29 years, for mandibular lesion ages ranged from 6 to 75 years, median 49.5 years and mean 46.16. Distribution of osteosarcomas according to age is depicted in Figure 1. 

**Figure 2:**
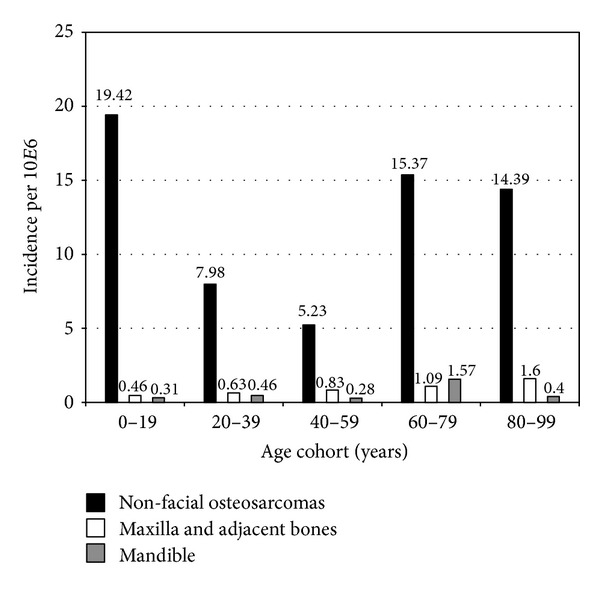
Age-corrected incidence per localization.

Age-corrected incidence rates were calculated on basis of 20-year age cohorts. These data were related to the distribution of the population in the year 2000. That year was selected since the year 2000 represents the median year of our cohort. In that year, 3.873.008 inhabitants of the Netherlands were 0–19 years of age, 4.761.504 were 20–39 years, 5.076.996 were 40–59 years, 1.652.103 were 60–79 years, and 500.339 were 80–99 years of age. Sites of involvement related to age categories are given in absolute numbers in Table 1.  Figure 2 provides data on age-corrected incidence per localization. Calculated age-specific incidences per million are given in Table 2. The highest percentages of jaw tumors were found in the three age categories from 20 to 79 years; 12.0, 17.4, and 14.8, percent, respectively. Whereas in younger (<20 years) and older patients (>79 years) only 3.9 and 2.0 percent of cases were jaw tumors (*P* < 0.001). In respect to histological type of tumors, the following categories were defined: conventional osteosarcoma (covering osteogenic as well as chondroblastic and fibroblastic osteosarcoma), telangiectatic osteosarcoma, secondary osteosarcoma related to Paget's disease, intraosseous low-grade osteosarcoma, parosteal osteosarcoma, periosteal sarcoma, and high-grade surface osteosarcoma. Distribution over the ages and various age-groups is given in Table 3.  Table 4 gives total numbers of non-gnatic, maxilla and mandible tumors according to histology; differences were significant (*P* = 0.01). Age-corrected incidence for histology figures is given in Table 5. Significances found were as follows: Paget's-related osteosarcoma was less frequent in ages < 60, and para-osteal osteosarcoma less prominent in the age range 0–19 and more frequent in the age range 20–39 (all *P* < 0.05). Assessing per age category the histological types in relation to localization significance was only found for conventional osteosarcoma, occurring more frequent in extragnatic sites in patients under the age of 20 years. But after correction for age-adjusted incidence, significance was lost. Modes of therapy are depicted in Table 6. After correction for age-specific incidence significantly more treatments with chemotherapy only were given below the age of 60 years, and only radiotherapy was given more frequent in patient above the age of 80 years, and only surgery was less done below the age of 40 years.  Table 7 gives data on therapy given per localization; differences were not significant (*P* = 0.10). Data on the analysis for tumors generally acknowledged to be in need for multimodality treatment; that is, conventional osteosarcomas, teleangiectatic osteosarcomas, and high-grade surface osteosarcomas are given in Table 8. After subanalysis per age group, patients <20 years of age more often received combination therapy (*P* < 0.001), and patients >80 years of age were treated with surgery only (*P* = 0.035). Additional separate analysis of conventional osteosarcomas for the separate age-groups revealed for nongnatic, maxilla and mandible following data: *P* < 0.000, *P* = 0.288, *P* = 0.128, respectively. For nonconventional osteosarcomas a *P* value of <0.000 was computed for nongnatic tumors; for other localizations numbers were too low. 

Higher percentages of older patients did not receive multimodality (surgery, chemotherapy, and/or radiotherapy) treatment (*P* < 0.05); single modality treatment is more often given to older patients as compared with younger individuals. In patients >79 years, the percentage of patients treated with only radiotherapy are substantially higher (*P* < 0.05). 

**Figure 3:**
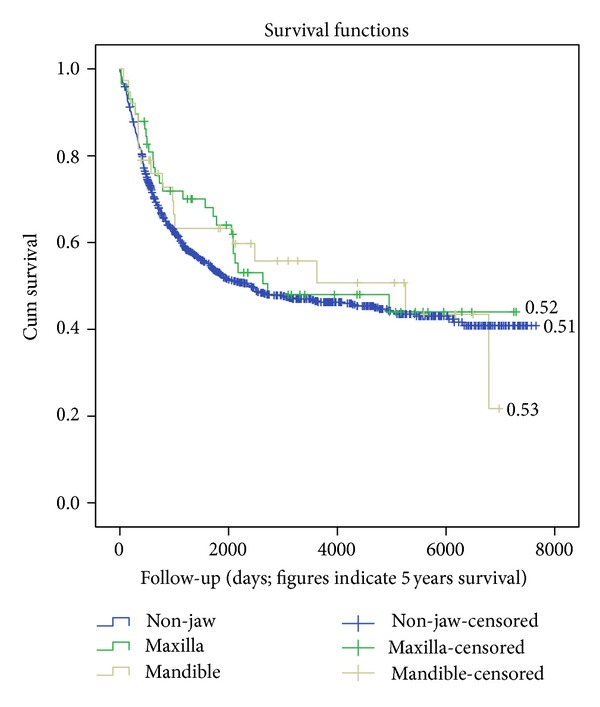
Overall survival according to localization.

**Figure 4:**
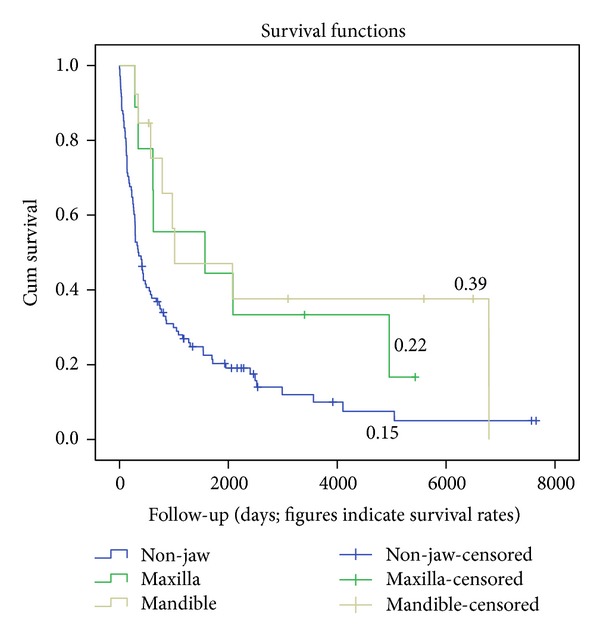
Overall survival in 60 to 79 year old persons.

From the database details on relapse and other events were incomplete. As a result no figures on event-free survival can be given. However, sound data on occurrence of death were available and overall survival rates (OS) could be computed. In respect to OS, no differences in gnatic versus nongnatic sites were found (Figure 3; log-rank 0.61). Assessment per age category revealed that patients aged between 60 to 79 years had a poorer outcome in case of extra-gnatic locations as compared to gnatic lesions (*P* = 0.031; Figure 4). 

**Figure 5:**
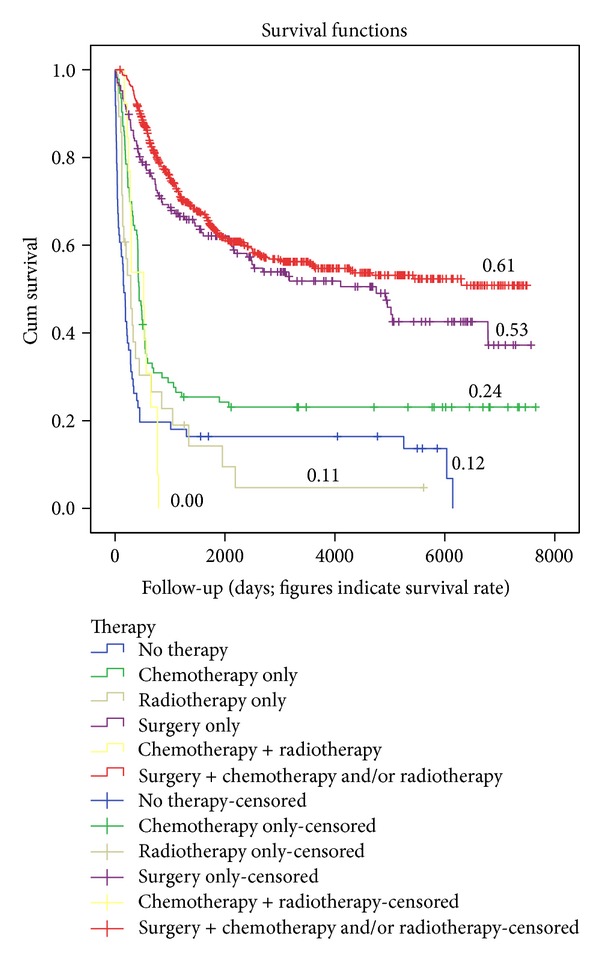
Overall survival in relation to therapy.

After sorting out the various pathology subsets also in the mentioned age-group only significance was found for conventional osteosarcomas (*P* = 0.013). Log-rank correlations revealed high significance for the various modes of treatment (*P* < 0.001). The highest survival rates were seen for combined modality treatment and surgery only versus the other modes of treatment. However, further analysis comparing combined modality treatment with surgery only was not significant (*P* = 0.084; see Figure 5).

Splitting it up into age categories only in the age category from 20 to 39 years, a significance was documented (*P* = 0.006). Analysis per age category revealed that with increasing age, overall survival showed a significant trend for poorer outcomes at older age (*P* < 0.001). Splitting the data up in nonjaw, maxilla, and mandible locations, significance for maxillary lesions was still prominent (*P* = 0.020) whilst the significance for mandible lesion overall survival in relation to age was lost, which might in part be due to the low numbers (see figure for non-gnatic lesions). Coxanalysis revealed that mode of therapy had the strongest influence on survival favoring multimodality treatment.

## 4. Discussion

Osteosarcomas are mostly noted in the long bones, and it is assumed that they have the highest incidence in the second decade of life. Although the number of craniofacial osteosarcomas is very low, the prevalence of jaw osteosarcoma is in fact 10 times greater than that of osteosarcoma in the total body skeleton, considering that jaws represent only 0.86% of total body volume [17]. International studies on osteosarcomas exclude craniofacial osteosarcomas based on the general assumption that these tumors have a histological lower malignancy grading and have a lower tendency for metastatic spread. Collecting data from the literature a number of problems arose if comparisons with our data were made. In the literature, many reports originate from single institutions or from, sometimes, voluntary registries. A summary of the reports that were found is depicted in Table 9 [1–4, 6–8, 10–12, 14–34]. These reports mentioned in total 1382 cases. The number of patients per report ranges from 7 to 496, with a mean of 45 cases per report. Three other problems hamper assessment of our data. The histopathological diagnosis is taken “as read” and probably there has been no histopathological review of several cases in this study. We were also not able to discern primary versus secondary tumors, except for those patients with Paget's disease of the bone as underlying disease. But this condition was not diagnosed in the mandible or maxilla in our group. Also, in the various reports from the literature, discrimination of primary osteosarcomas versus secondary disease was also not possible. 

Based on the distribution of ages in our cohort, it is likely that osteosarcomas of the jaws have a nonequal distribution of age-specific incidence across the age ranges. Generally, a peak incidence in the 4th and 5th decade of life is mentioned [1, 7, 12, 14, 16, 19]. This is in contrast to the Japanese data and the data from Bologna stating that the occurrence is similar across age groups, but unfortunately these data were not corrected for age [4, 24]. Based on our data a substantial increase of age-corrected incidence occurs after the age of 60 in non-gnatic as well as gnatic tumors. The absence of the peak in the first two decades of life is specific for gnatic tumors. In the older age ranges, a possible bias might have occurred due to previous radiotherapy for nonmalignant diseases in the head-neck region [35–37]. Due to the number of patients treated with radiotherapy and the decreasing dosages and smaller fields of irradiation currently used, it is likely that the number of secondary tumors will decrease in the coming decades. The lower percentage in older patients treated with multimodality treatment can be explained by the choice of both physicians and older patients to go for care instead of cure in these often frail patients [38–40]. Localization in the maxillary region exceeded mandible localizations, which is not in line with other publications [41]. A predominance in relation with gender was not noted in our cohort, which is also in contrast with some other reports mentioning either male or female but confirms data of others on the equal distribution among genders [6, 14, 15, 42]. The peculiar peak incidences noted in Japan of mandibular tumors in females only were not seen in our cohort [10, 24].

In respect to pathology grading, the current statement that craniofacial osteosarcomas are relatively benign cannot be confirmed from our data. The majority are conventional osteosarcomas, and the relative benign parosteal osteosarcoma was only found once in the jaws, whereas relative benign periosteal osteosarcoma was not diagnosed. However part of the statement on the mild character of these tumors is based on the assumed incidence of metastatic spread. We do not have data on distant metastatic lesions and later outcome in respect to recurrence. Reports in literature are contradictive. The finding that in only one cohort (60–79 years) the overall survival was significantly poorer for extra-gnatic tumors is not fully reassuring on a more benign behavior either, since they are occurring in an older population in which the baseline all-cause survival is lower. 

Since we have limited data on outcome and treatment modalities, we can make only limited statements. From our data some conclusions, can be made: (1) at an older age treatment is more often confined to surgery or radiotherapy, and from the age of 60 years onwards chemotherapy is less often given in malignant diseases. (2) Outcome decreases with increasing age. (3) No significant differences could be attributed to site of the osteosarcoma in the maxilla, mandible or extra-gnatic sites. (4) No significant differences in relation to age were found based on histology. Based on our data, the mode of treatment clearly favors multimodality treatment combining chemotherapy with surgery. For combination of chemotherapy and radiotherapy, no conclusions can be made due to the low frequency of radiotherapy, which is clearly related to the low efficacy of radiotherapy in osteosarcomas. In the literature, two meta-analyses gave opposite results in respect to the chemotherapy issue in jaw tumors. In the meta-analysis of Smeele et al., statistical analysis revealed that surgical margins and chemotherapy were independent significant factors for disease-free and overall survival and radiotherapy had an insignificant effect [43]. Kassir et al. mention a poorer survival in case adjuvant chemotherapy was given. This study might be biased as the authors have no data on surgical margins [44]. Other studies suggest a survival benefit if chemotherapy is added, which confirm our findings [8, 17]. Other prognostic factors such as completeness of resection and chemotherapy-induced tumor necrosis, which is the major prognostic factor in osteosarcomas in general, are not available for craniofacial tumors [45]. Size of the tumor and low-grade histology have been assumed to reflect a better prognosis [1, 14, 20]. 

We conclude that osteosarcomas have comparable incidences below the age of 20 years as compared with ages >60 years. Poorer outcome in older people might be due to refraining from chemotherapy. Treatment of both nonjaw and jaw osteosarcomas is in the far majority based on multimodality treatment composed of chemotherapy and surgery. 

## Figures and Tables

**Table 1:** Number of cases in relation to localization and age.

Number of cases	Nongnatic	Maxilla	Mandible
Age category			
0–19 year	367	9	6
20–39 year	190	15	11
40–59 year	133	21	7
60–79 year	127	9	13
80–99 year	36	4	1

**Table 2:** Age-corrected incidence rates per million.

Age category (years)	Total incidence	Extragnatic	Maxilla	Mandible
0–19	19.72	19.42	0.46	0.31
20–39	9.07	7.98	0.63	0.46
40–59	6.34	5.23	0.83	0.28
60–79	18.03	15.37	1.09	1.57
80–99	16.39	14.39	1.60	0.40

**Table 3:** Distribution over the various age groups.

	Conventional OS	Telangiectatic OS	Paget disease-related OS	Intraosteal low malignant OS	Paraosteal OS	Peri-osteal OS	High-grade surface OS
0–19 years							
*N* =	348	22	0	1	7	4	0
%	42.0%	48.9%	.0%	100.0%	12.3%	80.0%	.0%
20–39 years							
*N* =	180	9	0	0	26	1	0
%	21.7%	20.0%	.0%	.0%	45.6%	20.0%	.0%
40–59 years							
*N* =	137	7	1	0	15	0	1
%	16.5%	15.6%	9.1%	.0%	26.3%	.0%	100.0%
60–79 years							
*N* =	130	5	7	0	7	0	0
%	15.7%	11.1%	63.6%	.0%	12.3%	.0%	.0%
80–99 years							
*N* =	34	2	3	0	2	0	0
%	4.1%	4.4%	27.3%	.0%	3.5%	.0%	.0%

OS: osteosarcoma.

**Table 4:** Distribution of histology per localization.

	Non-jaw	Maxilla	Mandible
Conventional osteosarcoma	736	56	37
Telangiectatic osteosarcoma	44	0	1
Paget's disease-related osteosarcoma	11	0	0
Intraosseal low malignant osteosarcoma	0	1	0
Paraosteal osteosarcoma	56	1	0
Periosteal osteosarcoma	5	0	0
High-grade surface osteosarcoma	1	0	0

**Table 5:** Age-corrected incidence rates per million for histology.

Age category (years)	Conventional osteosarcoma	Telangiectatic osteosarcoma	Paget-related osteosarcoma	Intraosseal low malingant osteosarcoma	Paraosteal osteosarcoma	Periosteal osteosarcoma	High-grade surface osteosarcoma
0–19	17.97	0.93	0.00	0.05	0.36	0.21	0.00
20–39	7.56	0.32	0.00	0.00	1.09	0.04	0.00
40–59	5.40	0.21	25.38	0.00	0.59	0.00	0.04
60–79	15.74	1.91	57.82	0.00	0.85	0.00	0.00
80–99	13.59	5.43	7.51	0.00	0.80	0.00	0.00

**Table 6:** Modes of therapy.

	No therapy	Chemotherapy only	Radiotherapy only	Surgery only	Chemotherapy + radiotherapy	Surgery + chemotherapy and/or radiotherapy	Total
0–19 years							
*N* =	9	33	5	6	6	311	370
%	2.4%	8.9%	1.4%	1.6%	1.6%	84.1%	100.0%
20–39 years							
*N* =	3	27	1	22	2	134	189
%	1.6%	14.3%	.5%	11.6%	1.1%	70.9%	100.0%
40–59 years							
*N* =	9	14	4	27	3	88	145
%	6.2%	9.7%	2.8%	18.6%	2.1%	60.7%	100.0%
60–79 years							
*N* =	24	17	9	57	2	26	135
%	17.8%	12.6%	6.7%	42.2%	1.5%	19.3%	100.0%
80–99 years							
*N* =	10	0	8	12	0	6	36
%	27.8%	.0%	22.2%	33.3%	.0%	16.7%	100.0%

Total							
*N* =	55	91	27	124	13	565	875
%	6.3%	10.4%	3.1%	14.2%	1.5%	64.6%	100.0%

**Table 7:** Distribution of multimodality treatment per localization.

	Non-jaw	Maxilla	Mandible
Conventional osteosarcoma	480	30	14
Telangiectatic osteosarcoma	40	0	1
Paget's-disease related osteosarcoma	4	0	0
Intraosseal low malignant osteosarcoma	0	0	0
Paraosteal osteosarcoma	16	0	0
Periosteal osteosarcoma	2	0	0
High-grade surface osteosarcoma	0	0	0

**Table 8:** Therapy for tumors needing multimodality treatment.

Age	No therapy	Chemotherapy only	Radiotherapy only	Surgery only	Chemotherapy + radiotherapy	Surgery + chemotherapy and/or radiotherapy	Total
0–19 years							
*N* =	9	33	5	6	6	311	370
%	2.4%	8.9%	1.4%	1.6%	1.6%	84.1%	100.0%
20–39 years							
*N* =	3	27	1	22	2	134	189
%	1.6%	14.3%	.5%	11.6%	1.1%	70.9%	100.0%
40–59 years							
*N* =	9	14	4	27	3	88	145
%	6.2%	9.7%	2.8%	18.6%	2.1%	60.7%	100.0%
60–79 years							
*N* =	24	17	9	57	2	26	135
%	17.8%	12.6%	6.7%	42.2%	1.5%	19.3%	100.0%
80–99 years							
*N* =	10	0	8	12	0	6	36
%	27.8%	.0%	22.2%	33.3%	.0%	16.7%	100.0%

Total							
*N* =	55	91	27	124	13	565	875
%	6.3%	10.4%	3.1%	14.2%	1.5%	64.6%	100.0%

**Table 9:** Manuscripts in the literature specific for gnatic osteosarcomas.

Report	Origin of the data	Total number of patients	Number of primary tumors	Number of patients with underlying bone disease	Percentage of tumors expressed on total of tumors located in the yaws	
					Mandible	Maxilla
Oda et al. [8]	SI	13	9	2	62	38
Vege et al. [10]	SI	34	34	0	64	36
van Es et al. [18]	MI	46	37	7	49	51
Delgado et al. [2]	SI	28	27	1	48	52
Padilla and Murrah [19]	SI	7	7	0	71	29
Caron et al. [6]	SI	43	29	11	53	47
Mark et al. [20]	SI	18	14	4	67	33
Gadwal et al. [3]	SR	22	22	0	95	5
Junior et al. [21]	SI	24	21	0	63	37
Huh et al. [22]	SI	12	12	0	75	25
Lewis et al. [23]	SI	12	9	1	41	59
Clark et al. [14]	SI	66	59	2	49	51
Forteza et al. [12]	SI	9	9	0	44	56
Bertoni et al. [4]	SI	28	27	0	71	29
Doval et al. [1]	SI	8	NR		50	50
Tanzawa et al. [24]	MI	114	NR		59	41
Slootweg and Muller [16]	SI	18	17	0	44	56
Daw et al. [34]	SI	18	10		40	60
Can Soc OLHNSOSG	MR	35	NR		57	43
August et al. [15]	MR	30	27	0	57	43
Smith et al. [25]	SR	496	NR		47	53
Nissanka et al. [17]	SI	19	NR		58	42
Bennett et al. [26]	SI	25	16	1	70	30
McHugh et al. [27]	SI	21	15	0	50	50
Fernandes et al. [28]	SI	16	13	2	56	44
Jasnau et al. [29]	SR	49	36		56	44
Guadagnolo et al. [30]	SI	62	NR		53	47
Huber et al. [31]	MI	14	8	2	50	50
Garrington et al. [7]	SR	56	51	2	68	32
Ha et al. [32]	SR	27	20	2	46	54
Thiele et al. [33]	SI	12	NR		42	58

S: single, M: multiple, I: institution, and R: registry.
